# Supplementary material for: Anderson-Darling and Watson tests for the geometric distribution with estimated probability of success
Source: PLoS One. 2024 Dec 31;19(12):e0315855. doi: 10.1371/journal.pone.0315855 (PMC11687809; doi:10.1371/journal.pone.0315855)
Supplement: Attachment — Submitted filename: RebbutalLetter.pdf [file pone.0315855.s001.pdf]

Universidad Veracruzana

Instituto de Investigaciones en Inteligencia Artificial

Región Xalapa

Campus Sur,
Calle Paseo Lote II,
Sección Segunda
N° 112, Nuevo
Xalapa,

Xalapa, Ver.
México 91097

Teléfono

Conmutador

8421700

Conm. Ext.

10201

www.uv.mx/iiia

Dear Professor Oluwafemi Samson Balogun, Ph. D.
Academic Editor
PLOS ONE

We extend our sincere appreciation for your dedicated time and effort as academic editor for our article, as well as for the diligent work of the referees who reviewed our paper.

Below, we summarize the changes and corrections made to our manuscript, along with our responses to the referees' feedback:

1) Our manuscript now adheres to PLOS ONE's style requirements, including file naming conventions.

2) The code and data used to construct analyzed runs is now available at the following website: https://doi.org/10.5281/zenodo.10659806

3) We have clarified the role of funders as follows: "The funders had no role in study design, data collection and analysis, decision to publish, or preparation of the manuscript."

4) Funding information has been removed from the Acknowledgments section of our manuscript.

5) Responses to reviewers' comments and suggestions:

Starting with referee number 1, we appreciate the positive feedback provided. In response, we have added a brief introduction to our paper highlighting various applications of the geometric distribution in hydrology, technology, high energy physics, etc., supported by relevant citations.

Regarding referee #2's comments:

“1. The paper needs to be proof-read for removal of spelling and grammatical errors.”

We have thoroughly proofread the entire manuscript to eliminate spelling and grammatical errors.

“2. There are some obvious improvements in the introductory and literature review parts of the manuscript.”

We have written a genuine introduction and reorganized this by adding two subsections as will be explained latter. Please refer to lines 2 to 56 of the revised paper.

With regard to the bibliography, it has been corrected and expanded; in addition, the articles that were not referenced in the text of the first version of the article have been removed.

For more details on it, see point “6)” and sub-items b), c), and d) below.

“3. Problem Statement needs to be improved.”

The Problem Statement has been enhanced and clarified and section introduction has been reorganized adding a subsection titled "1.1 Problem Statement". Please refer to lines 20 to 52 of the revised paper.

“4. Interpretation of the Table or data analysis part should be elaborated.”

We have elaborated on the interpretation of the table and data analysis by explicitly explaining all table variables within the paper text and table labels. Please, see sub-items g) and h) below and lines 143 to 152 of the new version of our paper and explanatory label of table number 5.

“5. Conclusion of the study should be elaborated, currently, it is merely a summary of the research. It is very important to pinpoint the implications of your study.”

The Conclusion section has been revised and rewritten to emphasize the implications of our findings, their applications, the limitations of our study and potential future research directions. See lines 165 to 209 of our paper.

“6. Delete the low-level journal references and non-scientific references from the paper.”

We have updated the reference list and highlighted all changes in the Revised Manuscript. We have also addressed concerns about low-level and non-scientific references, but we kindly request specific guidance on which references should be removed if necessary. We assume that reviewer #2 refers to those relate to the python programs used, where we do give credit to those whose work helped us to perform the presented calculations.

Below, we detail a summary of changes and corrections made.

a) The title of the article has been changed to provide a closer explanation of the topic being discussed.

The original title was "Cramer-von Mises tests for the geometric distribution with estimated probability of success," and the new title will be: "Anderson-Darling and Watson tests for the geometric distribution with estimated probability of success."

b) Section “1 Introduction” has been rewritten, indicating importance and applications of Geometric distributions with respective references. See lines 1 to 19.

c) Still in “Section 1 Introduction”, subsection “1,1 Problem statement” has been introduced. See lines 21 to 52.

d) Again in the introduction, a new section titles “1,2 Preliminary definitions” has been added to clarify text. Minor corrections to original text here included in lines 54 to 56

e) Although the labeling of the tables was correct, their presentation order was wrong, so this have been corrected in addition to that respective labels have been enhanced with a clearer explanation of them, specifically:

Labels of tables 1 and 2:

Addition of the phrase “...for different significance levels α” at the end of respective labels.

f) Labels of tables 3 and 4:

Addition of the phrase “...with different significance levels α” at the end of corresponding labels.

g) Label of table 5:

Before:

Table 3. Distribution of lengths of trends from series of returns and summary statistics.

Now:

Table 5. Distribution of lengths of trends and summary statistics for all analyzed markets.

a) Observed runs duration; b) total number of runs; c) observed average run length; d) θ estimated; e) 95% C.I. for θ; f) and g) estimated values of W 2 and A2 statistics, respectively, with corresponding p−values.

Also, table 5 has now an additional column, the first, to make a better explanation of it.

h) Explanation of table 5 has been added in text. See lines 143 to 152.

i) As required by referee #2, section "Conclusion" has been revised and rewritten, mentioning the implications of our findings, their applications, the limitations of our study, and potential future research directions. See lines numbers 165 to 209.

Finally, many minor corrections have been made, which can be seen in the underlined version of our manuscript.

Dear Professor Oluwafemi, once again, we express our gratitude to you and our two reviewers for your valuable feedback and guidance throughout the review process.

Warm regards,

On behalf of all authors,

Dr. Alejandro Raúl Hernández Montoya
Universidad Veracruzana, México.
alhernandez@uv.mx
www.uv.mx/personal/alhernandez
