# Supplementary material for: Anderson-Darling and Watson tests for the geometric distribution with estimated probability of success
Source: PLoS One. 2024 Dec 31;19(12):e0315855. doi: 10.1371/journal.pone.0315855 (PMC11687809; doi:10.1371/journal.pone.0315855)
Supplement: Attachment — Submitted filename: PONE_D_23_22495.pdf [file pone.0315855.s002.pdf]

Referee's report : PONE-D-23-22495

Cramer-von Mises tests for the geometric distribution with estimated probability of success

Submitted for a possible publication in **PLOS ONE**

In this manuscript, tests of fit for the geometric distribution, utilizing the discrete version of the Watson and Anderson-Darling statistics are discussed. An moderately extensive tables containing the asymptotic percentage points for these test statistics, accompanied by illustrative examples drawn from the realm of Finance is presented. Specifically, the application of our statistical methodology to daily time series data of financial indices are demonstrated.

Some comments are as follows;

1. In Equation (1), a range of θ must be specified.
2. In Equations (2)-(7), what is Z_i?
3. When the `momentchi2` is employed [1], how to implement it?
4. It seems R is used in this study, it may be cited [2].
5. R source code may be provided.

References

[1] D. Bodenham. momentchi2: Moment-Matching Methods for Weighted Sums of Chi-Squared Random Variables, 2016. R package version 0.1.5.

[2] R Core Team. R: A Language and Environment for Statistical Computing. R Foundation for Statistical Computing, Vienna, Austria, 2023.
