# Supplementary material for: Anderson-Darling and Watson tests for the geometric distribution with estimated probability of success
Source: PLoS One. 2024 Dec 31;19(12):e0315855. doi: 10.1371/journal.pone.0315855 (PMC11687809; doi:10.1371/journal.pone.0315855)
Supplement: Attachment — Submitted filename: RebutalLetter062024.pdf [file pone.0315855.s003.pdf]

Dear Professor Oluwafemi Samson Balogun, Ph. D.
Academic Editor
PLOS ONE

Here are our responses to the comments and suggestions from referee number 4. Once again, we appreciate the time you and our referees have dedicated to evaluating our article.

1. In Equation (1), a range of θ must be specified.
The range $0 \leq \theta \leq 1$ has been specified, and the text "which gives the probability that the first success occurs on the ith trial." has been included. See lines 21 and 23 respectively of revised manuscript.

2. In Equations (2)-(7), what is Zi ?
We have included the following text in lines 60 to 62 of the revised paper:

By definition Zj is the cumulative sum of the differences between the observed frequencies o_i , and the theoretically expected frequencies e_i for all cells or categories from i =1 up to i = j.

3. When the momentchi2 is employed [1], how to implement it?

We utilized the library by calling its function Momentchi2, using the lpb4 functionality (Lindsay-Pilla-Basak method) within the context of the proposed analysis according to the official documentation, ensuring that necessary parameters were correctly passed and following recommended practices to be sure the routine runs correctly. We used the lpb4 option in the momentchi2 library because it offers a highly efficient and accurate method for computing the cumulative distribution function (CDF) for chi-squared distributions. This option is particularly suitable for our analysis as it establishes precise results with lower computational cost, which is crucial for handling large datasets and complex statistical models, especially when computer equipment is not last generation, such as is our case.

4. It seems R is used in this study, it may be cited [2].
We did not use R in our study, instead we have used python.

5. R source code may be provided.
We have provided python code. See https://zenodo.org/doi/10.5281/zenodo.10519808

Given its relevance to academics and others interested in these topics, we have cited reference [2] related to the R package and mentioned by referee No. 4 (cited as [24] in paper), reference [1] mentioned by our referee it is already mentioned since previous revised version of our paper (reference [22] in paper). Those references are:

[1] D. Bodenham. momentchi2: Moment-Matching Methods for Weighted Sums of Chi-Squared Random Variables, 2016. R package version 0.1.5.

[2] R Core Team. R: A Language and Environment for Statistical Computing. R Foundation for Statistical Computing, Vienna, Austria, 2023.

Dear Professor Oluwafemi, once again, we express our gratitude to you and all our reviewers for valuable feedbacks and guidance throughout the review process.

Warm regards,

On behal of of all authors,

Dr. Alejandro Raúl Hernández Montoya
Universidad Veracruzana,
alhernandez@uv.mx
www.uv.mx/personal/alhernandez
