# Supplementary material for: Anderson-Darling and Watson tests for the geometric distribution with estimated probability of success
Source: PLoS One. 2024 Dec 31;19(12):e0315855. doi: 10.1371/journal.pone.0315855 (PMC11687809; doi:10.1371/journal.pone.0315855)
Supplement: Attachment — Submitted filename: RebuttaLetterEditor.pdf [file pone.0315855.s004.pdf]

Xalapa, Veracruz. México. October/24/2024.

Dear Professor Ahmed Mahmoud Gemeay
Academic Editor
PLOS One

We have carefully addressed each of your comments and suggestions regarding our paper, and our detailed responses are provided below. We truly appreciate the time and effort you have dedicated to reviewing our manuscript, and we hope that our responses are clear and satisfactory.

I find this approach interesting, but more details on the new model should be added using the following significant comments.

1- What is the motivation and contribution of this paper? Authors must write two or three paragraphs in the introduction to show the novelty and importance of their paper.

We explained our motivation and contribution on the paper. To explain the motivation that led this paper we have included the following text in a subsection named "Motivation" in the introduction section:

"subsection{Motivation}
In our opinion, there is still work to be done in developing rigorous testing methods for assessing the goodness-of-fit for discrete models, particularly for the geometric distribution. While tests such as Anderson-Darling and Watson have been primarily developed for continuous distributions, relatively few alternatives have been adapted for discrete cases. This study aims to contribute to this issue by adapting the Anderson-Darling and Watson tests to a discrete model, in our case the geometric distribution and provide a valuable tool for researchers working with this data."

"Our motivation for this work stems from a previous investigation into the geometric distribution's application to the duration of "price runs" in financial indices. In our earlier research \cite{Hernandez}, we analyzed the behavior of price runs in daily financial indices such as the NASDAQ, DJIA, and Nikkei 225. Through this study, we identified the need for a rigorous statistical test that could better assess the fit of geometric distributions in real-world financial data. The results of that research emphasized the limitations of current goodness-of-fit tests for discrete models, prompting the development of the current study."

We have already described our contribution in the text or our paper:

"The difference between the above mentioned kind of tests and the tests presented in this paper, is that for the geometric case and from asymptotic theory of test statistics,

see section \ref{sec:AsymtoticTheory}, we calculate the tests statistics asymptotic distributions for W^{2} and A^{2}, and we give their respective Tables \ref{tableW2} and \ref{tableA2} for different values of the parameter θ. We also provide an explicit explanation of the procedure necessary to apply the geometric fit tests developed here, see section \ref{sec:TestProcedure}. We aim to test statistically the null hypothesis that the data sample to analyze was drawn from the geometric distribution. Finally, we are making the Python code needed to perform the corresponding calculations and tests available."

2 - Authors should add more recent papers discussing related problems and their relation to their paper if they exist.

Although we were able to find many papers on the geometric distribution, there are not many recent ones that discuss fitting it and describing a goodness-of-fit test. Instead, we found numerous papers on generalizing the geometric distribution, and one on generalizing and fitting it, which was the closest and recent reference we could cite, and its reference number is 20.

We also cite a work, although not recent, that serves as a precursor to ours. It presents goodness-of-fit tests for the geometric distribution based on discrete adaptations of the Anderson-Darling A^{2} test and others, and compares them with each other. Its reference number is 19.

3 - Rewrite the abstract again to be more attractive.

We appreciate your suggestion; the abstract has been modified in order to make it more attractive:

New abstract:
"We propose two novel goodness-of-fit tests for the geometric distribution based on discrete adaptations of the Watson W^{2} and Anderson-Darling A^{2} statistics, where the probability of success is unknown. Although these tests are widely applied to continuous distributions, their application in discrete models has been relatively unexplored. Our study addresses this need by developing a robust statistical framework specifically for discrete distributions, particularly the geometric distribution. We provide extensive tables of asymptotic critical values for these tests and demonstrate their practical relevance through a financial case study. Specifically, we apply these tests to analyze price runs derived from daily time series of NASDAQ, DJIA, Nikkei 225, and the Mexican IPC indices, covering the period from January 1, 2015, to December 31, 2022. This work broadens the range of available tools for assessing goodness-of-fit in discrete models, which are essential for applications in finance and beyond. The Python programs developed for this paper are available to the academic community."

4 - I need clarification as to why these two measures are new for discrete models; the authors should have explained them in the paper.

The measures are not new as we state in the text:

“This type of tests has already been constructed for some important discrete distributions such as the Poisson distribution (Spinelli and Stephens [1]), the firts-digit Benford distribution (Lesperance et al. [3]) and for the discrete uniform distribution (Choulakian, Lockhart and Stephens [2].)”

Our new contribution is the methodology presented, that lies in adapting these tests for use with the geometric model. The tests we present offer a robust approach for discrete models. we have calculated detailed asymptotic tables for these tests, which extend what has been previously available, particularly for discrete models. These tables provide critical values for a wide range of parameter settings, offering a valuable resource for researchers. Also, we present a novel application of these tests in the field of finance, specifically analyzing ‘price runs’ in financial indices. The study of price runs, a variable that reflects uninterrupted trends in market prices, has been relatively underexplored, making this a significant contribution to both statistical methodology and financial research. Moreover, we have made the programs developed for conducting these goodness-of-fit tests publicly available, ensuring that the academic community can easily replicate and extend our results.

5 - Authors used MLE to find their estimator; can any other estimation method be used? Can they add more studies on this estimator, such as unbiased property?

We use MLE because it is preferred over other estimation methods due to its consistency, asymptotic efficiency, asymptotic normality, and flexibility. Although other methods may be useful in specific contexts, MLE generally offers the best statistical properties under standard conditions.

We would like to clarify that our focus is on the presented goodness-of-fit tests rather than on the estimation methods themselves. Our tests can be applied regardless of whether the parameters are estimated using MLE, the Method of Moments, or any other estimation method biased or not. The performance and conclusions of the tests remain consistent, as they are designed to assess the goodness of fit between the observed data and the theoretical geometric distribution model.

6 - What symbol does infinity refer to in Tables 1 and 2? What size do authors use to write this symbol?

The infinity symbol (refers to the theoretically calculated values based on the asymptotic distribution, which is derived when the sample size approaches infinity.

Regarding the size of the infinity symbol in Tables 3 and 4, it is set to 10pt. LaTeX uses the default font size, which is 10pt.

We have introduced the following text in our paper to explain this in detail:

"In Tables 3 and 4, the notation of infinity (\infty) refers to the theoretically calculated values based on the asymptotic distribution, which represents the distribution of the test statistics W^2 and A^2 when the sample size n approaches infinity. These asymptotic values provide a theoretical benchmark derived from the limiting behavior of the statistics as the number of observations grows large."

7 - Can authors add another example from another field?

We appreciate your request for additional revisions, we understand the importance of maintaining high standards in the review process. However, we would like to kindly bring to your attention some challenges we are currently facing: During previous rounds of review, the process was delayed, as new referees were selected each time we submitted revisions. This caused some frustration among the authors, particularly as one of the authors was mistakenly invited to review their own paper three times. This led to two of our co-authors requesting to withdraw the manuscript, although we were able to convince them otherwise, which also required additional time.

Given these circumstances, we respectfully ask if it would be possible to move forward with the current version of the manuscript.

Thank you very much for your understanding and support. We remain fully committed to delivering a high-quality contribution to the journal and appreciate all the effort invested in the review process.

8 - The English language should be revised.
English has been revised and errors corrected.

9 - All equations must be revised to see if they need a comma, a point, or a number at the end of them.

All equations have been revised, and those requiring numbering have been numbered. Additionally, other punctuation and minor typos have been corrected.

Once again, we sincerely thank you for your time and thoughtful attention in reviewing our manuscript.

On behalf of all authors.

Dr. Alejandro Raúl Hernández Montoya
Instituto de Investigaciones en IA
Universidad Veracruzana
Xalapa, Veracruz. México
alhernandez@uv.mx
www.uv.mx/personal/alhernandez
